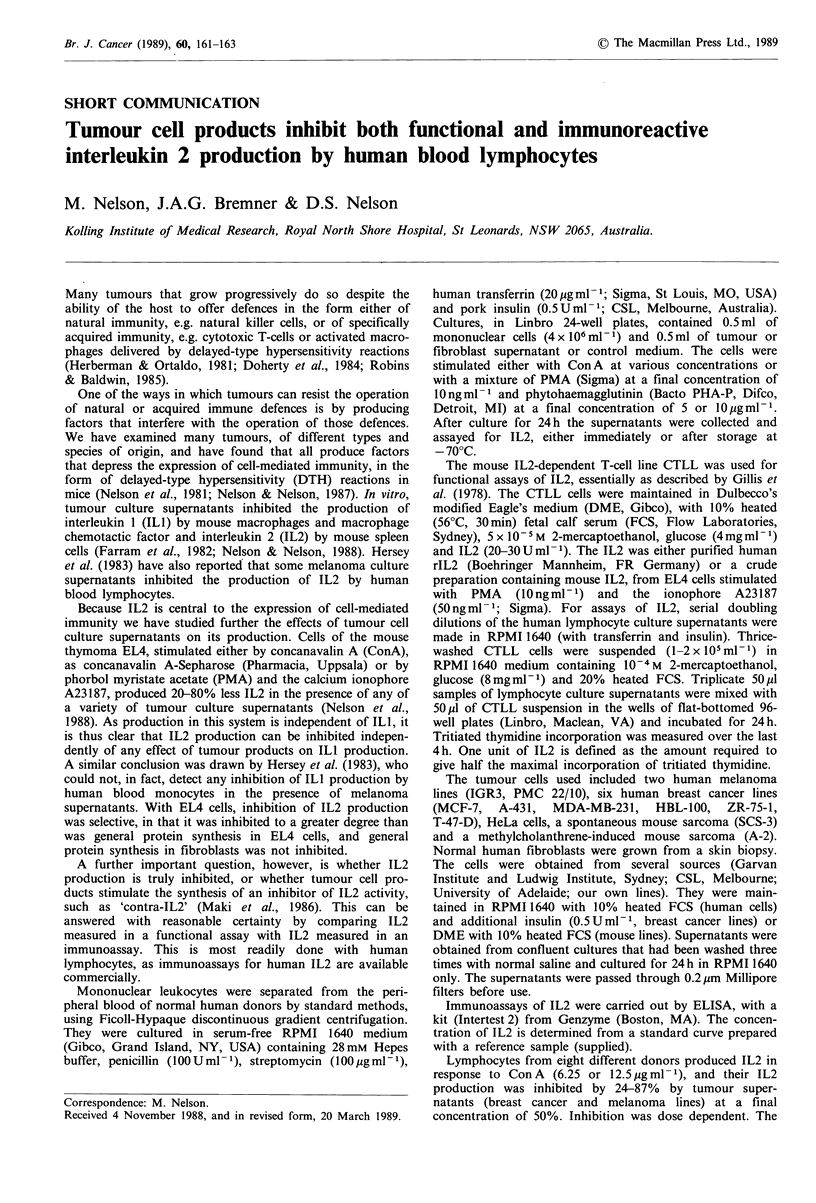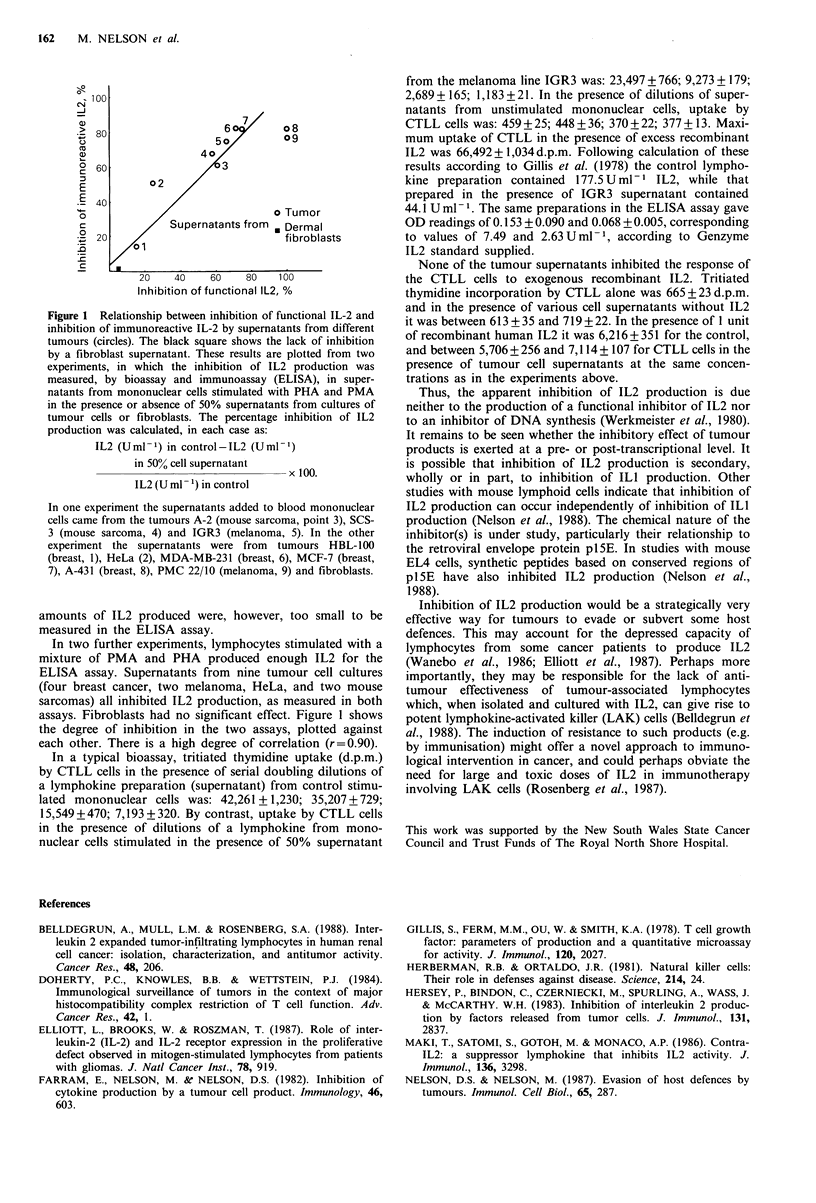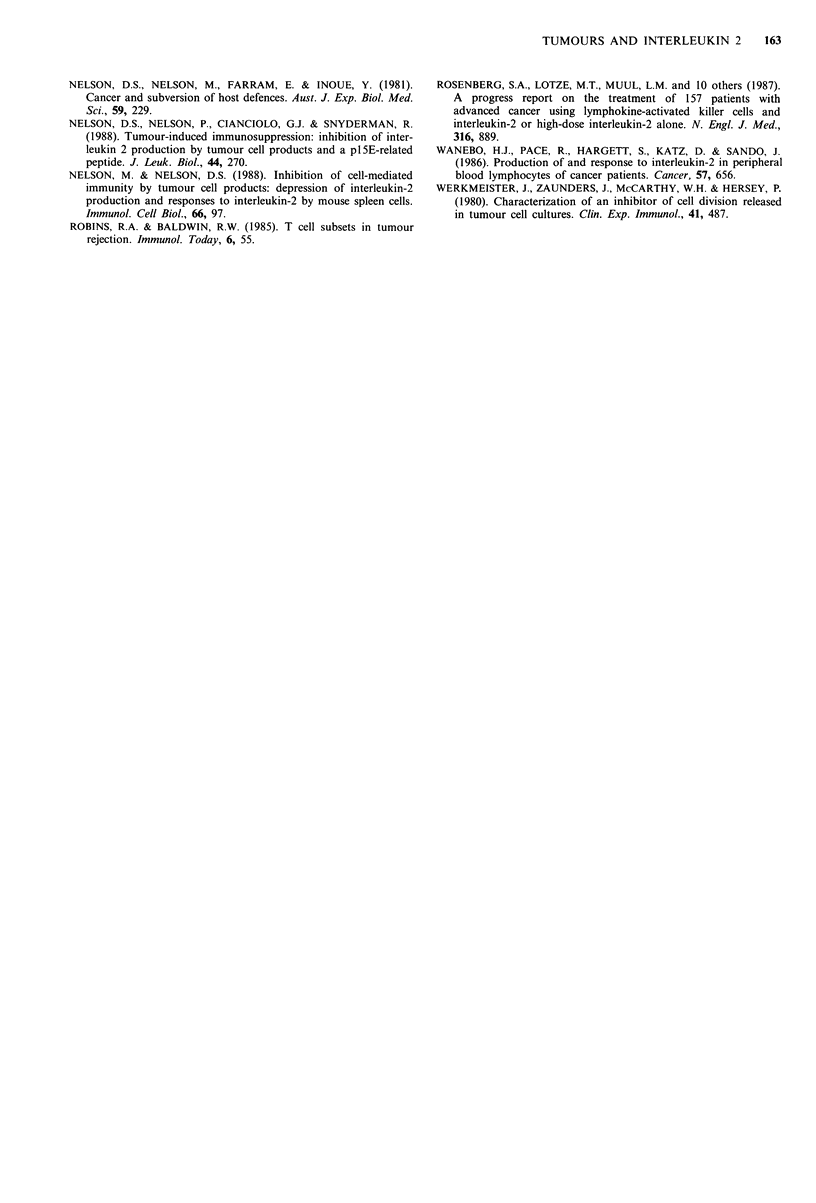# Tumour cell products inhibit both functional and immunoreactive interleukin 2 production by human blood lymphocytes.

**DOI:** 10.1038/bjc.1989.243

**Published:** 1989-08

**Authors:** M. Nelson, J. A. Bremner, D. S. Nelson

**Affiliations:** Kolling Institute of Medical Research, Royal North Shore Hospital, St Leonards, Australia.


Br. J. Cancer (1989), 60, 161–163 © The Macmillan Press Ltd., 1989

SHORT COMMUNICATION

### Tumour cell products inhibit both functional and immunoreactive interleukin 2 production by human blood lymphocytes

M. Nelson, J.A.G. Bremner & D.S. Nelson

*Kolling Institute of Medical Research, Royal North Shore Hospital, St Leonards, NSW 2065, Australia.*

Many tumours that grow progressively do so despite the ability of the host to offer defences in the form either of natural immunity, e.g. natural killer cells, or of specifically acquired immunity, e.g. cytotoxic T-cells or activated macrophages delivered by delayed-type hypersensitivity reactions (Herberman & Ortaldo, 1981; Doherty *et al.*, 1984; Robins & Baldwin, 1985).

One of the ways in which tumours can resist the operation of natural or acquired immune defences is by producing factors that interfere with the operation of those defences. We have examined many tumours, of different types and species of origin, and have found that all produce factors that depress the expression of cell-mediated immunity, in the form of delayed-type hypersensitivity (DTH) reactions in mice (Nelson *et al.*, 1981; Nelson & Nelson, 1987). *In vitro*, tumour culture supernatants inhibited the production of interleukin 1 (IL1) by mouse macrophages and macrophage chemotactic factor and interleukin 2 (IL2) by mouse spleen cells (Farram *et al.*, 1982; Nelson & Nelson, 1988). Hersey *et al.* (1983) have also reported that some melanoma culture supernatants inhibited the production of IL2 by human blood lymphocytes.

Because IL2 is central to the expression of cell-mediated immunity we have studied further the effects of tumour cell culture supernatants on its production. Cells of the mouse thymoma EL4, stimulated either by concanavalin A (ConA), as concanavalin A-Sepharose (Pharmacia, Uppsala) or by phorbol myristate acetate (PMA) and the calcium ionophore A23187, produced 20–80% less IL2 in the presence of any of a variety of tumour culture supernatants (Nelson *et al.*, 1988). As production in this system is independent of IL1, it is thus clear that IL2 production can be inhibited independently of any effect of tumour products on IL1 production. A similar conclusion was drawn by Hersey *et al.* (1983), who could not, in fact, detect any inhibition of IL1 production by human blood monocytes in the presence of melanoma supernatants. With EL4 cells, inhibition of IL2 production was selective, in that it was inhibited to a greater degree than was general protein synthesis in EL4 cells, and general protein synthesis in fibroblasts was not inhibited.

A further important question, however, is whether IL2 production is truly inhibited, or whether tumour cell products stimulate the synthesis of an inhibitor of IL2 activity, such as 'contra-IL2' (Maki *et al.*, 1986). This can be answered with reasonable certainty by comparing IL2 measured in a functional assay with IL2 measured in an immunoassay. This is most readily done with human lymphocytes, as immunoassays for human IL2 are available commercially.

Mononuclear leukocytes were separated from the peripheral blood of normal human donors by standard methods, using Ficoll-Hypaque discontinuous gradient centrifugation. They were cultured in serum-free RPMI 1640 medium (Gibco, Grand Island, NY, USA) containing 28 mM Hepes buffer, penicillin ($100\,U\,ml^{-1}$), streptomycin ($100\,\mu g\,ml^{-1}$), human transferrin ($20\,\mu g\,ml^{-1}$; Sigma, St Louis, MO, USA) and pork insulin ($0.5\,U\,ml^{-1}$; CSL, Melbourne, Australia). Cultures, in Linbro 24-well plates, contained 0.5 ml of mononuclear cells ($4\times10^{6}\,ml^{-1}$) and 0.5 ml of tumour or fibroblast supernatant or control medium. The cells were stimulated either with Con A at various concentrations or with a mixture of PMA (Sigma) at a final concentration of $10\,ng\,ml^{-1}$ and phytohaemagglutinin (Bacto PHA-P, Difco, Detroit, MI) at a final concentration of 5 or $10\,\mu g\,ml^{-1}$. After culture for 24 h the supernatants were collected and assayed for IL2, either immediately or after storage at −70°C.

The mouse IL2-dependent T-cell line CTLL was used for functional assays of IL2, essentially as described by Gillis *et al.* (1978). The CTLL cells were maintained in Dulbecco's modified Eagle's medium (DME, Gibco), with 10% heated (56°C, 30 min) fetal calf serum (FCS, Flow Laboratories, Sydney), $5\times10^{-5}$ M 2-mercaptoethanol, glucose ($4\,mg\,ml^{-1}$) and IL2 ($20–30\,U\,ml^{-1}$). The IL2 was either purified human rIL2 (Boehringer Mannheim, FR Germany) or a crude preparation containing mouse IL2, from EL4 cells stimulated with PMA ($10\,ng\,ml^{-1}$) and the ionophore A23187 ($50\,ng\,ml^{-1}$; Sigma). For assays of IL2, serial doubling dilutions of the human lymphocyte culture supernatants were made in RPMI 1640 (with transferrin and insulin). Thrice-washed CTLL cells were suspended ($1–2\times10^{5}\,ml^{-1}$) in RPMI 1640 medium containing $10^{-4}$ M 2-mercaptoethanol, glucose ($8\,mg\,ml^{-1}$) and 20% heated FCS. Triplicate $50\,\mu l$ samples of lymphocyte culture supernatants were mixed with $50\,\mu l$ of CTLL suspension in the wells of flat-bottomed 96-well plates (Linbro, Maclean, VA) and incubated for 24 h. Tritiated thymidine incorporation was measured over the last 4 h. One unit of IL2 is defined as the amount required to give half the maximal incorporation of tritiated thymidine.

The tumour cells used included two human melanoma lines (IGR3, PMC 22/10), six human breast cancer lines (MCF-7, A-431, MDA-MB-231, HBL-100, ZR-75-1, T-47-D), HeLa cells, a spontaneous mouse sarcoma (SCS-3) and a methylcholanthrene-induced mouse sarcoma (A-2). Normal human fibroblasts were grown from a skin biopsy. The cells were obtained from several sources (Garvan Institute and Ludwig Institute, Sydney; CSL, Melbourne; University of Adelaide; our own lines). They were maintained in RPMI 1640 with 10% heated FCS (human cells) and additional insulin ($0.5\,U\,ml^{-1}$, breast cancer lines) or DME with 10% heated FCS (mouse lines). Supernatants were obtained from confluent cultures that had been washed three times with normal saline and cultured for 24 h in RPMI 1640 only. The supernatants were passed through $0.2\,\mu m$ Millipore filters before use.

Immunoassays of IL2 were carried out by ELISA, with a kit (Intertest 2) from Genzyme (Boston, MA). The concentration of IL2 is determined from a standard curve prepared with a reference sample (supplied).

Lymphocytes from eight different donors produced IL2 in response to Con A (6.25 or $12.5\,\mu g\,ml^{-1}$), and their IL2 production was inhibited by 24–87% by tumour supernatants (breast cancer and melanoma lines) at a final concentration of 50%. Inhibition was dose dependent. The

Correspondence: M. Nelson.
Received 4 November 1988, and in revised form, 20 March 1989.

**Figure 1** Relationship between inhibition of functional IL-2 and inhibition of immunoreactive IL-2 by supernatants from different tumours (circles). The black square shows the lack of inhibition by a fibroblast supernatant. These results are plotted from two experiments, in which the inhibition of IL2 production was measured, by bioassay and immunoassay (ELISA), in supernatants from mononuclear cells stimulated with PHA and PMA in the presence or absence of 50% supernatants from cultures of tumour cells or fibroblasts. The percentage inhibition of IL2 production was calculated, in each case as:

$$\frac{\text{IL2 (U ml}^{-1}\text{) in control} - \text{IL2 (U ml}^{-1}\text{) in 50\% cell supernatant}}{\text{IL2 (U ml}^{-1}\text{) in control}} \times 100.$$

In one experiment the supernatants added to blood mononuclear cells came from the tumours A-2 (mouse sarcoma, point 3), SCS-3 (mouse sarcoma, 4) and IGR3 (melanoma, 5). In the other experiment the supernatants were from tumours HBL-100 (breast, 1), HeLa (2), MDA-MB-231 (breast, 6), MCF-7 (breast, 7), A-431 (breast, 8), PMC 22/10 (melanoma, 9) and fibroblasts.

amounts of IL2 produced were, however, too small to be measured in the ELISA assay.

In two further experiments, lymphocytes stimulated with a mixture of PMA and PHA produced enough IL2 for the ELISA assay. Supernatants from nine tumour cell cultures (four breast cancer, two melanoma, HeLa, and two mouse sarcomas) all inhibited IL2 production, as measured in both assays. Fibroblasts had no significant effect. Figure 1 shows the degree of inhibition in the two assays, plotted against each other. There is a high degree of correlation ($r = 0.90$).

In a typical bioassay, tritiated thymidine uptake (d.p.m.) by CTLL cells in the presence of serial doubling dilutions of a lymphokine preparation (supernatant) from control stimulated mononuclear cells was: 42,261 ± 1,230; 35,207 ± 729; 15,549 ± 470; 7,193 ± 320. By contrast, uptake by CTLL cells in the presence of dilutions of a lymphokine from mononuclear cells stimulated in the presence of 50% supernatant from the melanoma line IGR3 was: 23,497 ± 766; 9,273 ± 179; 2,689 ± 165; 1,183 ± 21. In the presence of dilutions of supernatants from unstimulated mononuclear cells, uptake by CTLL cells was: 459 ± 25; 448 ± 36; 370 ± 22; 377 ± 13. Maximum uptake of CTLL in the presence of excess recombinant IL2 was 66,492 ± 1,034 d.p.m. Following calculation of these results according to Gillis *et al.* (1978) the control lymphokine preparation contained 177.5 U ml$^{-1}$ IL2, while that prepared in the presence of IGR3 supernatant contained 44.1 U ml$^{-1}$. The same preparations in the ELISA assay gave OD readings of 0.153 ± 0.090 and 0.068 ± 0.005, corresponding to values of 7.49 and 2.63 U ml$^{-1}$, according to Genzyme IL2 standard supplied.

None of the tumour supernatants inhibited the response of the CTLL cells to exogenous recombinant IL2. Tritiated thymidine incorporation by CTLL alone was 665 ± 23 d.p.m. and in the presence of various cell supernatants without IL2 it was between 613 ± 35 and 719 ± 22. In the presence of 1 unit of recombinant human IL2 it was 6,216 ± 351 for the control, and between 5,706 ± 256 and 7,114 ± 107 for CTLL cells in the presence of tumour cell supernatants at the same concentrations as in the experiments above.

Thus, the apparent inhibition of IL2 production is due neither to the production of a functional inhibitor of IL2 nor to an inhibitor of DNA synthesis (Werkmeister *et al.*, 1980). It remains to be seen whether the inhibitory effect of tumour products is exerted at a pre- or post-transcriptional level. It is possible that inhibition of IL2 production is secondary, wholly or in part, to inhibition of IL1 production. Other studies with mouse lymphoid cells indicate that inhibition of IL2 production can occur independently of inhibition of IL1 production (Nelson *et al.*, 1988). The chemical nature of the inhibitor(s) is under study, particularly their relationship to the retroviral envelope protein p15E. In studies with mouse EL4 cells, synthetic peptides based on conserved regions of p15E have also inhibited IL2 production (Nelson *et al.*, 1988).

Inhibition of IL2 production would be a strategically very effective way for tumours to evade or subvert some host defences. This may account for the depressed capacity of lymphocytes from some cancer patients to produce IL2 (Wanebo *et al.*, 1986; Elliott *et al.*, 1987). Perhaps more importantly, they may be responsible for the lack of anti-tumour effectiveness of tumour-associated lymphocytes which, when isolated and cultured with IL2, can give rise to potent lymphokine-activated killer (LAK) cells (Belldegrun *et al.*, 1988). The induction of resistance to such products (e.g. by immunisation) might offer a novel approach to immunological intervention in cancer, and could perhaps obviate the need for large and toxic doses of IL2 in immunotherapy involving LAK cells (Rosenberg *et al.*, 1987).

This work was supported by the New South Wales State Cancer Council and Trust Funds of The Royal North Shore Hospital.

### References

BELLDEGRUN, A., MULL, L.M. & ROSENBERG, S.A. (1988). Interleukin 2 expanded tumor-infiltrating lymphocytes in human renal cell cancer: isolation, characterization, and antitumor activity. *Cancer Res.*, **48**, 206.

DOHERTY, P.C., KNOWLES, B.B. & WETTSTEIN, P.J. (1984). Immunological surveillance of tumors in the context of major histocompatibility complex restriction of T cell function. *Adv. Cancer Res.*, **42**, 1.

ELLIOTT, L., BROOKS, W. & ROSZMAN, T. (1987). Role of interleukin-2 (IL-2) and IL-2 receptor expression in the proliferative defect observed in mitogen-stimulated lymphocytes from patients with gliomas. *J. Natl Cancer Inst.*, **78**, 919.

FARRAM, E., NELSON, M. & NELSON, D.S. (1982). Inhibition of cytokine production by a tumour cell product. *Immunology*, **46**, 603.

GILLIS, S., FERM, M.M., OU, W. & SMITH, K.A. (1978). T cell growth factor: parameters of production and a quantitative microassay for activity. *J. Immunol.*, **120**, 2027.

HERBERMAN, R.B. & ORTALDO, J.R. (1981). Natural killer cells: Their role in defenses against disease. *Science*, **214**, 24.

HERSEY, P., BINDON, C., CZERNIECKI, M., SPURLING, A., WASS, J. & McCARTHY. W.H. (1983). Inhibition of interleukin 2 production by factors released from tumor cells. *J. Immunol.*, **131**, 2837.

MAKI, T., SATOMI, S., GOTOH, M. & MONACO, A.P. (1986). Contra-IL2: a suppressor lymphokine that inhibits IL2 activity. *J. Immunol.*, **136**, 3298.

NELSON, D.S. & NELSON, M. (1987). Evasion of host defences by tumours. *Immunol. Cell Biol.*, **65**, 287.

NELSON, D.S., NELSON, M., FARRAM, E. & INOUE, Y. (1981). Cancer and subversion of host defences. *Aust. J. Exp. Biol. Med. Sci.*, **59**, 229.

NELSON, D.S., NELSON, P., CIANCIOLO, G.J. & SNYDERMAN, R. (1988). Tumour-induced immunosuppression: inhibition of interleukin 2 production by tumour cell products and a p15E-related peptide. *J. Leuk. Biol.*, **44**, 270.

NELSON, M. & NELSON, D.S. (1988). Inhibition of cell-mediated immunity by tumour cell products: depression of interleukin-2 production and responses to interleukin-2 by mouse spleen cells. *Immunol. Cell Biol.*, **66**, 97.

ROBINS, R.A. & BALDWIN, R.W. (1985). T cell subsets in tumour rejection. *Immunol. Today*, **6**, 55.

ROSENBERG, S.A., LOTZE, M.T., MUUL, L.M. and 10 others (1987). A progress report on the treatment of 157 patients with advanced cancer using lymphokine-activated killer cells and interleukin-2 or high-dose interleukin-2 alone. *N. Engl. J. Med.*, **316**, 889.

WANEBO, H.J., PACE, R., HARGETT, S., KATZ, D. & SANDO, J. (1986). Production of and response to interleukin-2 in peripheral blood lymphocytes of cancer patients. *Cancer*, **57**, 656.

WERKMEISTER, J., ZAUNDERS, J., McCARTHY, W.H. & HERSEY, P. (1980). Characterization of an inhibitor of cell division released in tumour cell cultures. *Clin. Exp. Immunol.*, **41**, 487.